# Prognostic significance of multinucleated giant cells in oral squamous cell carcinoma: A retrospective clinicopathological study

**DOI:** 10.1016/j.jobcr.2025.12.005

**Published:** 2025-12-16

**Authors:** Kuldeep Singh, Priya Kumar, Aadithya B. Urs

**Affiliations:** Department of Oral Pathology, Maulana Azad Institute of Dental Sciences, New Delhi, India

**Keywords:** Bryne's grading system, Clinico-pathological parameters, Multinucleated giant cell, Oral squamous cell carcinoma, Overall survival, Prognosis

## Abstract

**Background:**

Multinucleated Giant Cells (MGCs) are akin to immune cells that play a crucial role in the tumor microenvironment. The present study aimed to evaluate the prognostic and clinicopathological significance of MGCs in Oral Squamous Cell Carcinoma (OSCC) through comprehensive analysis of their presence, localization, and correlation with survival outcomes.

**Objective:**

To determine whether the presence and distribution of MGCs in OSCC correlates with tumor stage, histopathological features, and overall survival, thereby assessing their potential prognostic value.

**Material and methods:**

100 proven cases of OSCC were retrieved with complete clinicopathological data. Haematoxylin-eosin sections were reviewed independently by three pathologists and grouped into two, with and without MGCs. The distribution, and average number of MGCs per 5HPF per case were examined. All clinicopathological parameters and prognosis based on Bryne's grading were compared between groups. The overall survival with follow-up period of >36 months was analyzed.

**Results:**

34 cases with MGCs were found, out of which 21 patients are alive. Cases with MGCs had pTNM stage III whereas majority of cases without MGC's had stage IV. Significant correlation was found for lympho-vascular invasion only. Cases with MGC's showed good prognostic score according to Bryne's grading, while without MGC's showed moderate to poor prognostic score. Cases of alveolar mucosa, including gingiva and ridge SCC with MGCs, showed poor survival.

**Conclusions:**

This study highlights the potential role of MGCs in OSCC, suggesting an association with earlier tumor stages and favorable prognostic scores. Further research is needed to assess their prognostic and therapeutic relevance.

## 1 Introduction

Head and Neck Squamous Cell Carcinoma includes oral cavity tumors, oropharynx, hypopharynx, nasopharynx, and larynx. In India, Oral Squamous Cell Carcinoma (OSCC) is leading malignancy, with most patients presenting at a locally advanced stage at the time of diagnosis. OSCC represents approximately 80–90 % of all malignant neoplasms of the oral cavity. The risk factors for the development of OSCC include cigarette smoking, alcohol consumption, betel quid chewing, inadequate nutrition, poor oral hygiene, HPV and Epstein-Barr virus infections, and *Candida albicans* infections.^1^^,^^2^

The complex milieu of extracellular matrix elements and non-cancerous cells known as the Tumor Microenvironment (TME) surrounds solid tumors, and it is the reciprocity of these relationships that ultimately determines the malignant behavior of the tumor.^3^

In the TME, there are both innate cells [macrophages, dendritic Cells, lymphocytes, and Natural Killer cells and adaptive (T and B cell) immune cells, with CD8+T cells, NKs, and DCs playing the most efficient anti-tumor roles.^4^

Among the many inflammatory cells seen in the TME, macrophages are one of the most prominent components in many cancers, including OSCC.^4^ Macrophages have two distinct cell populations because of a mechanism known as macrophage polarisation, in which the macrophage expresses different functional programmes in response to microenvironmental signals, acquiring a specific phenotype such as M1 or M2. The pro-inflammatory cytokines interleukin IL-1, IL-6, IL-12, and Tumour Necrosis Factor alpha are produced by M1 macrophages (classically activated macrophages), and these M1 macrophages can exhibit an antitumoral response in cancer. However, M2 macrophages (alternatively activated macrophages) may exhibit a pro-tumoral response by secreting IL-4, IL-10 cytokines, Transforming Growth Factor-beta, Vascular Endothelial Growth Factor, Matrix Metallo-Proteinases and CD163 antigen.^5^, ^6^, ^7^

Th1 and Th2 cytokines induce the fusion of macrophages and result in their differentiation into Multinucleated Giant Cells (MGCs). This has been characterised as a host response to tumoral cells in numerous forms of carcinomas, although the prognostic implications remain unclear.^8^, ^9^, ^10^, ^11^, ^12^

MGCs are commonly associated with keratin scavenging. Recent studies suggest that they have a more complex role in tumor biology. MGCs have been detected within tumor islands and in chemotherapy-treated cases, indicating their possible association with TME changes rather than solely as a reaction to keratin. Some cases even exhibit true tumor giant cells expressing epithelial markers, suggesting a distinct oncogenic behavior.^13^

Gessain G. et al. identified MGCs in HNSCC tumors, associating them with a favorable prognosis in both treatment-naive and preoperative chemotherapy-treated patients. Their increased density post-therapy suggests a role in the antitumoral response. Spatial transcriptomic and proteomic analysis revealed an MGC-specific signature resembling TREM2-expressing macrophages in keratin-rich tumor niches. These findings position MGCs as a potential prognostic biomarker.^14^

While the role of tumor-associated macrophages (TAMs) in OSCC progression is well-established, with M2-polarized macrophages linked to poor prognosis, the prognostic significance of MGCs remains unclear. Some studies suggest MGCs may not contribute to tumor progression, yet emerging evidence indicates a potential link between MGC presence and aggressive tumor behavior, irrespective of histological grade. If further validated, MGCs could be incorporated into histological grading and explored as potential therapeutic targets for improving patient outcomes.^13^

The nature and importance of MGC reactions in intraoral SCC is poorly understood. Data available implies that MGC reactions are likely the result of M2 macrophages and indicate a foreign body reaction to keratinized SCC cells.^15^^,^^16^

A recent study explored the potential relationship between MGC reactions and tumor progression in Oral Tongue Squamous Cell Carcinoma, which suggest that the absence of MGC reactions may serve as an indicator of more aggressive tumor behavior and progression in OTSCC, underlining the potential prognostic value of MGCs in assessing the aggressiveness of the disease.^17^

The present study was undertaken to comprehensively investigate the role of MGCs in OSCC and their possible prognostic implications. Specifically, the objectives were to:

1. Evaluate the presence and distribution (superficial and deep) of MGCs in histopathological sections of OSCC.
2. Assess the correlation between MGC presence and key clinicopathological parameters including TNM stage, depth of invasion (DOI), worst pattern of invasion (WPOI), lymphovascular invasion (LVI), and perineural invasion (PNI).
3. Compare prognostic scores and overall survival outcomes between cases with and without MGCs.
4. Determine the potential prognostic role of MGCs in predicting tumor behavior and patient outcomes.

## 2 Material and methods

One-hundred cases of OSCC obtained from the archives of Maulana Azad Institute of Dental Sciences, New Delhi, India were selected for the study. Cases were retrieved from departmental archives between 2010 and 2020, and all patients had a uniform follow-up period of ≥36 months to allow survival analysis. This study included OSCC cases that had undergone complete surgical resection and had paraffin blocks with sufficient tissue for histological examination. Sample-size estimation was performed using G∗Power 3.1 software (effect size 0.3, α = 0.05, power = 0.8), yielding a minimum of 92 cases; 100 cases were ultimately included to improve statistical robustness.

### 2.1 Histological classification and grading

Histological diagnosis of OSCC was performed according to the criteria outlined in the 5th Edition (2024) of the World Health Organization Classification of Head and Neck Tumors (IARC Press, Lyon).^18^ WHO grading was used for diagnostic classification, while Bryne's (1992) grading system was separately applied for prognostic evaluation of the invasive front, focusing on cellular morphology, invasion pattern, and stromal response. This distinction eliminates any methodological overlap between diagnostic and prognostic grading systems.

Inclusion criteria:

• Histopathologically confirmed OSCC with adequate paraffin-embedded tissue blocks.
• Completely excised lesions with full clinicopathological data.
• Minimum follow-up ≥ 36 months.
Exclusion criteria:
• Prior chemo-/radiotherapy.
• Incomplete clinical data or inadequate tissue for analysis.

This study was approved by the institutional ethical research committee wide No.F./18/81/MAIDS/Ethical Committee/2023/1290 dated 16th June 2022.

### 2.2 Microscopic evaluation of MGCs

All H&E-stained sections were examined under a Nikon Eclipse Ci-L microscope (Tokyo, Japan) for the presence (Group I) or absence (Group II) of MGCs.

**Fig. 1:**
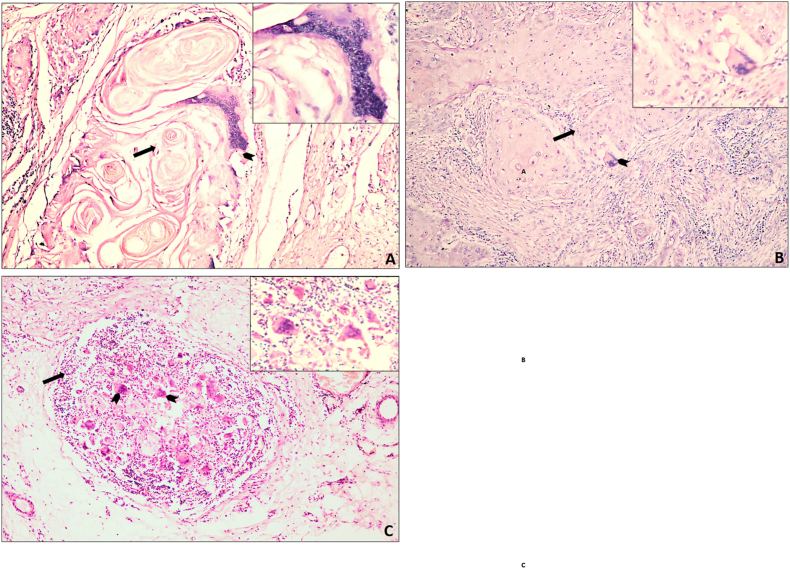
MGCs associated with keratin pearls (A), tumor cells (B), and inflammatory cells (C) in oral squamous cell carcinoma (OSCC). Images at 10 × ; insets at 40 × . Straight arrows indicate MGCs; arrowheads mark keratin pearl in (A), tumor islands in (B), and inflammatory cells in (C).

MGCs were identified according to the morphological criteria described by Brooks et al. (2009)^11^ (Fig. 1)

i. Located within, adjacent to the neoplastic cells and/or associated with foreign body material i.e, keratin in connective tissue.
ii. The cytoplasm should be glossy dense eosinophilic.
iii. Presence of at least three regularly scattered nuclei in the cytoplasm.

### 2.3 Quantification of MGCs

Each H&E-stained section was examined under a Nikon Eclipse Ci-L microscope at 400 × magnification. MGCs were identified using the morphological criteria of Brooks et al. (2009). For each case, five high-power fields (HPFs) showing the greatest MGC density were selected, and the average number of MGCs per 5 HPFs was recorded. MGCs were further categorized as superficial (adjacent to the epithelial–stromal interface) or deep (near skeletal muscle bundles) following Sánchez-Romero et al. (2018).^19^ The mean MGC count per case was calculated by dividing the total number by five.

### 2.4 Tumor staging and histopathological parameters

The eighth edition of the American Joint Committee on Cancer (AJCC) TNM staging system was used for tumor staging. Histopathological prognostic scoring was evaluated using the system proposed by Bryne et al. (1992).^20^^,^^21^ Additional histologic variables—WPOI, DOI, LVI, and PNI—were assessed following Brandwein-Gensler et al. (2005).^22^

### 2.5 Interobserver reliability

Two oral pathologists independently assessed all slides for the presence of MGCs. Cohen's Kappa coefficient (κ = 0.78) indicated substantial agreement, interpreted per Landis and Koch (1977)^23^ and the updated methodological guidance of McHugh (2012).^24^

### 2.6 Statistical analysis

All data were analyzed using IBM SPSS version 26. Categorical variables were compared using the Chi-square or Fisher's exact test; continuous variables were analyzed with the independent *t*-test. Overall survival was assessed using the Kaplan–Meier method with log-rank comparison. A p-value <0.05 was considered statistically significant. All p-values in Table 1, Table 2 and text have been re-verified for internal consistency.

**Table 1:** Distribution of OSCC cases according to clinico-pathological parameters in two groups: MGCs and without MGCs.

Clinico-pathological Parameters	with MGCs % (N = 34)	without MGCs % (N = 66)	P-value
**Age range (in yrs)**	29–91	28–84	
**Gender**			
Male	79.4 (27)	80.3 (53)	
Female	20.6 (7)	19.7 (13	
**Pathological TNM stage**			***0.037∗***
Stage I	14.7 (5)	4.5 (3)	
Stage II	20.6 (7)	19.7 (13)	
Stage III	38.2 (13)	19.7 (13)	
Stage IV (a+b)	26.5 (9)	56.1 (37)	
**DOI**			0.161
<10 mm	61.8 (21)	47 (31)	
**≥** 10 mm	38.2 (13)	53 (35)	
**WPOI (Grade)**			0.134
**1**	0 (0)	1.5 (1)	
**2**	2.9 (1)	3.0 (2)	
**3**	2.9 (1)	1.5 (1)	
**4**	5.9 (2)	6.1 (4)	
**1**–**4**	44.1 (15)	51.5 (34)	
**5**	44.1 (15)	36.4 (24)	
**L-V invasion**			***0.038∗***
Present	11.8 **(**4)	9 (6)	
Absent	88.2 (30)	91 (60)	
**PNI**			0.228
Present	38.2 **(**13)	21.2 (14)	
Absent	61.8 **(**14)	78.8 (52	
**Prognostic score**			***0.033∗***
Good	82 **(**28)	56 **(**37)	
Moderate	14.7 **(**5)	36.7 **(**24)	
Poor	2.9 **(**1)	7.6 **(**5)	
**Overall survival**			0.217
Alive	61.8 **(**21)	54.5 (36)	
Death	38.2 **(**13)	45.5 **(**30)	

OSCC, Oral Squamous cell carcinoma; MGC, Multinucleated giant cell; PNI, Perineural invasion; L-V invasion, Lymphovascular invasion; DOI, Depth of invasion; WPOI, Worst pattern of invasion.

**Table 2:** Distribution (based on site and location) of MGCs and correlate with overall survival.

Parameters	% (N = 34)	Overall Survival		P-value
		Alive	Death	
**Site**				***0.001∗***
Alveolar mucosa	14.7 (5)	0 (0)	100 (5)	
Buccal mucosa	41.2 (14)	78.6 (11)	21.4 (3)	
Tongue	44.1 (15)	66.7 (10)	33.3 (5)	
**Location**				0.581
Superficial	64.7 (22)	59 (13)	41 (9)	
Deep	29.4 (10)	60 (6)	40 (4)	
Superficial + Deep	5.9 (2)	100 (2)	0 (0)	
**With MGCs**	34	61.76 (21)	38.23 (13)	
**Avg. MGCs/case**		1.9	2.0	0.265
Min.		0.6	0.2	
Max.		6	4	
Mean (S.D)		1.990 (1.224)	2.046 (1.339)	

## 3 Results

### 3.1 Demographic and clinicopathological profile

Among 100 OSCC cases, 34 (34 %) exhibited MGCs, whereas 66 (66 %) lacked them. The mean age in the MGC-positive group was 60 years (range: 29–91) compared to 56 years (range: 28–84) in the MGC-negative group. The male-to-female ratio was 3.8:1 for MGC-positive cases and 2:1 for MGC-negative cases. A significant correlation was observed for pathological TNM stage (p = 0.037), indicating that MGC-positive cases were more frequently detected in earlier stages (I–II). Lymphovascular invasion (p = 0.038) also correlated significantly with MGC presence, while no significant associations were found with DOI, PNI, or WPOI grade (Table 1).

### 3.2 Histopathological prognostic score and MGC correlation

Based on Bryne's histopathological prognostic scoring, a significant difference (p = 0.033) was observed, with the majority of MGC-positive cases showing good prognosis. No significant correlation was found between the presence of MGCs and tumor differentiation grade (p = 0.284).

### 3.3 Overall survival analysis

Kaplan–Meier analysis showed better overall survival in MGC-positive cases (61.8 % alive) than in MGC-negative cases (54.5 % alive), though this difference was not statistically significant (p = 0.217).

**Fig. 2:**
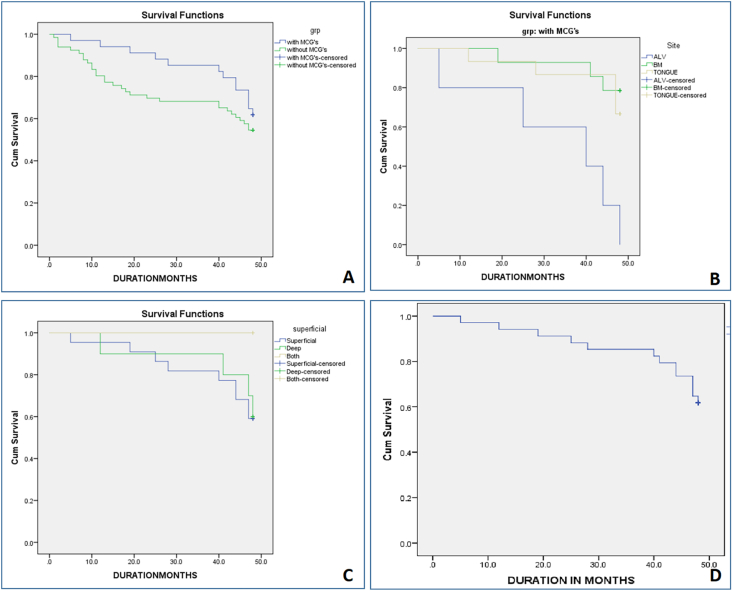
Kaplan-Meier Survival Analysis showing the correlation of overall survival with—(A) two group, with MGCs and without MGCs, (B) Lesion site distribution, (C) MGCs histologically depth-wise distribution in the tumor microenvironment, and (D) Overall average MGC count.

The Cohen's Kappa coefficient (κ = 0.78) confirmed strong interobserver reliability for MGC identification (Fig. 2A, Table 1).

### 3.4 Site-specific survival correlation

Among MGC-positive cases, anatomical site showed a highly significant correlation with survival (p = 0.001). All alveolar mucosa cases (gingiva and ridge) exhibited poor survival, while buccal mucosa and tongue lesions demonstrated improved outcomes (Table 2, Fig. 2B).

### 3.5 Depth-wise and quantitative analysis

The microscopic localization of MGCs (superficial, deep, or superficial + deep) did not significantly correlate with overall survival (p = 0.581). The mean number of MGCs per case ranged from 0.2 to 6 (mean ± SD: 2.01 ± 1.25). No statistical difference in mean MGC count was noted between survivors and non-survivors (p = 0.265) (Fig. 2C and D, Table 2).

## 4 Discussion

OSCC presents a major clinical challenge, and the tumor microenvironment (TME) plays a crucial role in its progression and prognosis. The TME plays a crucial role in OSCC pathogenesis and prognosis, and among its immune components, MGCs have emerged as a distinct but poorly characterized element.^1^, ^2^, ^3^, ^4^, ^5^, ^6^, ^7^

In this study, MGCs were observed in approximately 1/3rd of the OSCC cases, indicating their presence as a distinct histological feature within OSCC tumors. This finding is consistent with previous studies emphasising the heterogeneous nature of OSCC histopathology.^17^ Notably, this study revealed a significant correlation between the presence of MGCs and earlier pathological TNM stages (I and II) compared to cases without MGCs, which were predominantly observed in later stages (stage IV). This pattern suggests that MGCs may be associated with reduced tumor aggressiveness or invasiveness.^4^

We also found that LVI was significantly correlated with the presence of MGCs (*p* = 0.038). However, PNI did not show a significant correlation with MGCs presence (*p* = 0.288). These findings suggest that the presence of MGCs in OSCC cases may significantly influence the likelihood of LVI but not PNI.

Our study aligns with the findings of Gessain et al. (2024),^14^ who observed that the presence of MGCs in OSCC tumors was linked to earlier pathological TNM stages (I and II), suggesting a less aggressive phenotype. However, Gessain et al. also reported a positive correlation between MGC density and improved survival outcomes (overall survival and progression-free interval). In the present study, although a better survival outcomes was seen in cases with MGCs, these findings were not statistically significant (*p* = 0.217). This discrepancy may be due to differences in cohort size or patient demographics.

The presence of MGCs in tongue OSCC was associated with improved overall survival, with 66.7 % of cases surviving compared to 33.3 % in those without MGCs. This suggests a more favorable prognosis for patients with MGCs in OSCC of tongue, which aligns with the clinical significance of lesion location. Previous studies have shown that the absence of MGCs in oral tongue SCC is linked to tumor progression, advanced clinical stage, regional lymph node metastasis, and poorly differentiated tumors, all of which correlate with worse prognosis. Hence, the role of MGCs in its TME is further reinforced with its potential prognostication.^17^ However, few studies have suggested associations between immune cell infiltration, including MGCs or TAMs, and tumor invasion in OSCC.^4^^,^^6^ This variation may reflect methodological differences across studies and the inherent complexity of tumor–immune interactions.

The association between MGCs presence and different prognostic scores according to Bryne's system suggests a potential prognostic value of MGCs in OSCC. Our study demonstrated a higher proportion of “Good” prognoses in OSCC cases with MGCs, indicating a potential favorable prognostic influence of MGCs. However, further longitudinal analyses are warranted to validate these findings and delineate the precise prognostic significance of MGCs in OSCC.

**Within the 36-month follow-up, mortality was lower in MGC-positive (29 %) than in MGC-negative (45 %) cases, suggesting a possible survival advantage despite lack of statistical significance**. Previous studies have demonstrated association between immune cell infiltration, including TAMs, and survival outcomes in OSCC.^5^^,^^6^^,^^14^ This difference, although not statistically significant, may relate to limited sample size and patient heterogeneity. Analysis of Table 2 further illustrates the site-specific distribution of MGCs and their association with survival across the TME.

The data reveals notable variations in the prevalence of MGCs across different anatomical sites, with lesion on the buccal mucosa exhibiting the highest overall survival (78.6 %). The high prevalence of MGCs, particularly in cases with better patient survival rates, suggests a potential beneficial role of these cells in OSCC. Conversely, lesion on alveolar mucosa including gingiva and alveolar ridge presents a striking scenario with 100 % mortality, suggesting aggressive tumor behavior and potential challenges in therapeutic management because of early involvement of bone.

These findings partially agree with earlier studies suggesting that MGCs may derive from tumor-associated macrophages (TAMs) and reflect immune modulation within the TME, warranting further exploration of their prognostic relevance.^13^

Analyzing the distribution of MGCs based on depth within the tumor microenvironment reveals intriguing patterns. While the majority of cases exhibit superficial MGCs involvement, both superficial and deep locations showed comparable survival rates. This pattern likely reflects complex tumor–stroma interactions and the heterogeneous distribution of immune activity within the tumor mass.

Although the presence of MGCs alone does not exhibit a significant association with survival, differences in mean MGC count between survivors and non-survivors suggest a potential prognostic value that should be tested in larger cohorts.^1^^,^^12^ Understanding the distribution and impact of MGCs in OSCC holds significant implications for risk stratification and therapeutic decision-making. Gessain et al. further reported a correlation between tumor-infiltrating lymphocytes and MGC density, an aspect not analyzed here but relevant for future immunologic investigation.^14^

Overall, our findings indicate that MGCs are linked to early-stage OSCC, lower vascular invasion, and favorable prognostic parameters, supporting their potential as markers of host immune modulation rather than tumor aggression.

### 4.1 Strengths of the study

This study represents one of the few comprehensive analyses correlating MGCs with clinicopathological features and overall survival in OSCC. The use of dual histopathological systems — WHO 2024 for diagnostic classification and Bryne's 1992 grading for prognostic assessment — ensured methodological robustness and reproducibility. A relatively large, well-characterized cohort of 100 OSCC cases with a minimum 36-month follow-up provided reliable outcome data. Interobserver reliability (κ = 0.78) confirmed diagnostic consistency, and statistical correlations were independently validated using SPSS v26. Together, these factors strengthen the credibility of the findings and underscore the potential role of MGCs as a histopathological adjunct in prognostic evaluation.

### 4.2 Limitations of the study

The present study has certain limitations that must be acknowledged.

First, this was a retrospective, single-institutional study, which may limit the generalizability of the findings. Second, although morphological criteria were used for identifying MGCs, the absence of immunohistochemical validation (e.g., CD68, CD163 markers) prevents definitive confirmation of their macrophage origin. Third, the relatively small sample size, despite being statistically adequate, restricts subgroup analysis by tumor site or stage. Fourth, no correlation with molecular or immunological markers such as tumor-infiltrating lymphocytes (TILs) or cytokine profiles was performed, which could have provided deeper mechanistic insight.

Future studies with larger, multicentric cohorts incorporating immunohistochemical and molecular profiling are required to validate and expand upon these preliminary observations.

## 5 Conclusion

In conclusion, this study underscores the emerging significance of MGCs as a distinctive histological feature in OSCC. To the best of our knowledge, no previous studies have fully evaluated MGCs in OSCC in relation to histological parameters and overall survival. Our findings suggest that MGCs in OSCC are associated with earlier tumor stages and favorable prognostic scores, indicating a potentially less aggressive tumor phenotype. However, while no direct link to overall survival was found, the distribution of MGCs in OSCC across anatomical sites suggests their potential influence on tumor progression and patient outcomes.

The question remains: Do MGCs impact prognosis and survival in OSCC? While our study points to a possible association with less aggressive behavior, further research is required to confirm this. Future studies should involve larger, more diverse cohorts and explore the functional roles of MGCs in OSCC progression. Investigating their interaction with immune markers, like TILs, could provide deeper insights into OSCC's immunological mechanisms. Longitudinal studies are essential to evaluate the prognostic value of MGCs and their potential therapeutic target, ultimately enhancing OSCC management and patient outcomes.

## Patient consent statement

Since this study is observational in nature and does not involve direct patient intervention or identifiable personal data, patient's/guardian's consent was not required.

## Ethical considerations

This study involved the use of archived histopathological samples and anonymized clinical data from human subjects. Ethical approval was obtained from the Institutional Ethics Committee of Maulana Azad Institute of Dental Sciences, New Delhi (Approval No. F.18/81/MAIDS/Ethical Committee/2023/990; dated June 16, 2023), following submission of the revised study protocol (Version 2, dated June 10, 2023).

All procedures were conducted in accordance with the ethical standards of the institutional research committee, the 1964 Helsinki Declaration and its later amendments, and applicable national laws and guidelines.

As the study was retrospective in nature and did not involve any direct patient interaction or intervention, the requirement for written informed consent was waived by the Ethics Committee. No identifiable patient data were included in the study, and all records were anonymized to protect privacy and confidentiality. Appropriate measures were taken to ensure that the data used does not allow patient identification.

## Authors' contributions

Dr. Priya Kumar and Dr. Aadithya B. Urs collaboratively conceptualized the idea for the study. Dr. Kuldeep Singh collected the cases from the archival data. Dr. Priya Kumar and Dr. Aadithya B. Urs approved the archival cases. The initial analysis of the cases was performed independently by Dr. Kuldeep Singh and further approved independently by Dr. Priya Kumar and Dr. Aadithya B. Urs. Dr. Kuldeep Singh conducted the statistical analysis and drafted the manuscript. Dr. Priya Kumar and Dr. Aadithya B. Urs reviewed and approved the final manuscript.

## Source of funding

This research has not received specific funding from public sector agencies, commercial sector entities, or non-profit organisations.

## Declaration of competing interest

The authors declare that they have no conflicts of interest.
